# Recent advances in ferroptosis and therapeutic strategies for glioblastoma

**DOI:** 10.3389/fmolb.2022.1068437

**Published:** 2023-01-13

**Authors:** Qixiong Lu, Xiaoyang Lu, Yuansheng Zhang, Wei Huang, Hu Zhou, Tao Li

**Affiliations:** ^1^ The Affiliated Hospital of Kunming University of Science and Technology, Department of Neurosurgery, The First People's Hospital of Yunnan Province, Kunming, Yunnan, China; ^2^ Department of Neurosurgery, The First People’s Hospital of Yunnan Province, The Affiliated Hospital of Kunming University of Science and Technology, Kunming, Yunnan, China

**Keywords:** ferroptosis, glioblastoma, iron metabolism, chemotherapy, radiotherapy, immunotherapy

## Abstract

Ferroptosis is an emerging form of cell death characterized by the over-accumulation of iron-dependent lipid peroxidation. Ferroptosis directly or indirectly disturbs glutathione peroxidases cycle through diverse pathways, impacting the cellular antioxidant capacities, aggravating accumulation of reactive oxygen species in lipid, and it finally causes oxidative overload and cell death. Ferroptosis plays a significant role in the pathophysiological processes of many diseases. Glioblastoma is one of the most common primary malignant brain tumors in the central nervous system in adults. Although there are many treatment plans for it, such as surgical resection, radiotherapy, and chemotherapy, they are currently ineffective and the recurrent rate is almost up to 100%. The therapies abovementioned have a strong relationship with ferroptosis at the cellular and molecular level according to the results reported by numerous researchers. The regulation of ferroptosis can significantly determine the outcome of the cells of glioblastoma. Thus ferroptosis, as a regulated form of programed cell death, has the possibility for treating glioblastoma.

## Introduction

Ferroptosis is an emerging form of regulatable programed cell death compared with apoptosis, necroptosis, and pyroptosis [(Zhang et al., 2021a); (Shi et al., 2022a); (Zhou et al., 2019); (Bertheloot et al., 2021); (Li and Huang, 2022)]. In 2012, Dixon et al. named this regulatable cell death mode which could be inhibited by iron chelating agent as iron death on the basis of summarizing previous studies on cell deaths (Dixon et al., 2012). It is a metabolic process (Xu et al., 2021), not an active one that requires transcription induction or post-translational modification by specific death effectors in response to lethal stimuli. On the contrary, it is considered to be part of cell destruction or cell clearance.

Different from the previously described cell death modes, ferroptosis showed necrosis-like morphology: shrunk mitochondria, increased membrane density and narrowed crista, and ruptured cell membrane, whereas morphological changes were not evident in the nucleus; this kind of programmed cell death is initiated by abnormal metabolism of iron ions in cells, which causes the breakdown of the steady state of oxidoreduction reaction. The inhibition of the synthesis of reduced glutathione (GSH) and the intensification of oxidation reaction are the main causes of ferroptosis. A series of reactions that cause ferroptosis are iron dependent, triggered by intracellular changes in iron metabolism, leading to lipid peroxidation and finally cell death. (Lu et al., 2017; Mou et al., 2020; Aguilera et al., 2022). This mechanism is involved in pathophysiological conditions (Tang et al., 2018), including inflammation, tissue injuries, and cancerous transformation (Zhang et al., 2021a), among which glioblastoma (GBM) is one.

GBM, known for its devastating progression and dismal prognosis (Martínez-Garcia et al., 2018), is the most common primary malignant brain tumor in adults and accounts for over 50% of all high-grade gliomas (Martínez-Garcia et al., 2018). Its main characteristics are high malignancy, aggressive invasion, frequent brain or spinal cord metastasis and inevitable recurrence (Gu et al., 2016). According to the WHO grading system, GBM is classified as a Grade IV astrocytoma consisting of mostly low differentiated neoplastic astrocytes mixed with cells having different extent of differentiation (Ou-Yang et al., 2018) This is mainly due to the coexistence of multiple tumor cell populations with different degrees of differentiation, especially tumor cells that showing stem cell-like characteristics (Wang et al., 2019). Glioma stem cells have pretty strong renewal ability (Adamo et al., 2017), low differentiation, and high resistance to radiation and chemotherapy [(Broadley et al., 2011); (Sesé et al., 2022); (Liu et al., 2017)]. At present, there is no effective drug to treat it efficaciously, and surgery can only resect part of the tumor if it locates at functional or cranial base areas. Even if it can be removed completely with a negative intraoperative pathological edge, it virtually recurs through the mechanism we have not known yet. Therefore, the treatment scheme for recurrence can only remove the tumor to the maximum extent, and then carry out subsequent adjuvant chemotherapy and radiotherapy to destroy the tumor cells and inhibit the growth of tumor cells as much as possible (Berardinelli et al., 2019). However, the residual tumor cells are very easy to generate tolerance to radiotherapy and chemotherapy, and thus the recurrence rate of the tumor is as high as 100% [(Chanez et al., 2022); (Dissaux et al., 2021)]. After a series of changes in the internal structure of recurrent tumor cells, clonal evolution trees showed higher heterogeneity (Yang et al., 2020), and their malignancy is higher than the first time; that may be the reason why now no clinically available methods can effectively treat it. The average survival time of GBM patients after diagnosis is 12–15 months (Shi et al., 2015). It is urgent to develop molecular targeted drugs that are capable of effectively controlling the growth of GBM tumor cells based on their metabolic pathways.

Although few studies on the relationship between ferroptosis and GBM have been reported, especially using ferroptosis as a treatment or/and diagnostic method and the exploration of the relevant mechanism on cell death, recurrence and drug resistance between the two. We believe it is valuable to discuss this issue and it is what we attempted to deliver through this review. 

## Iron metabolism affects the ferroptosis

**FIGURE 1:**
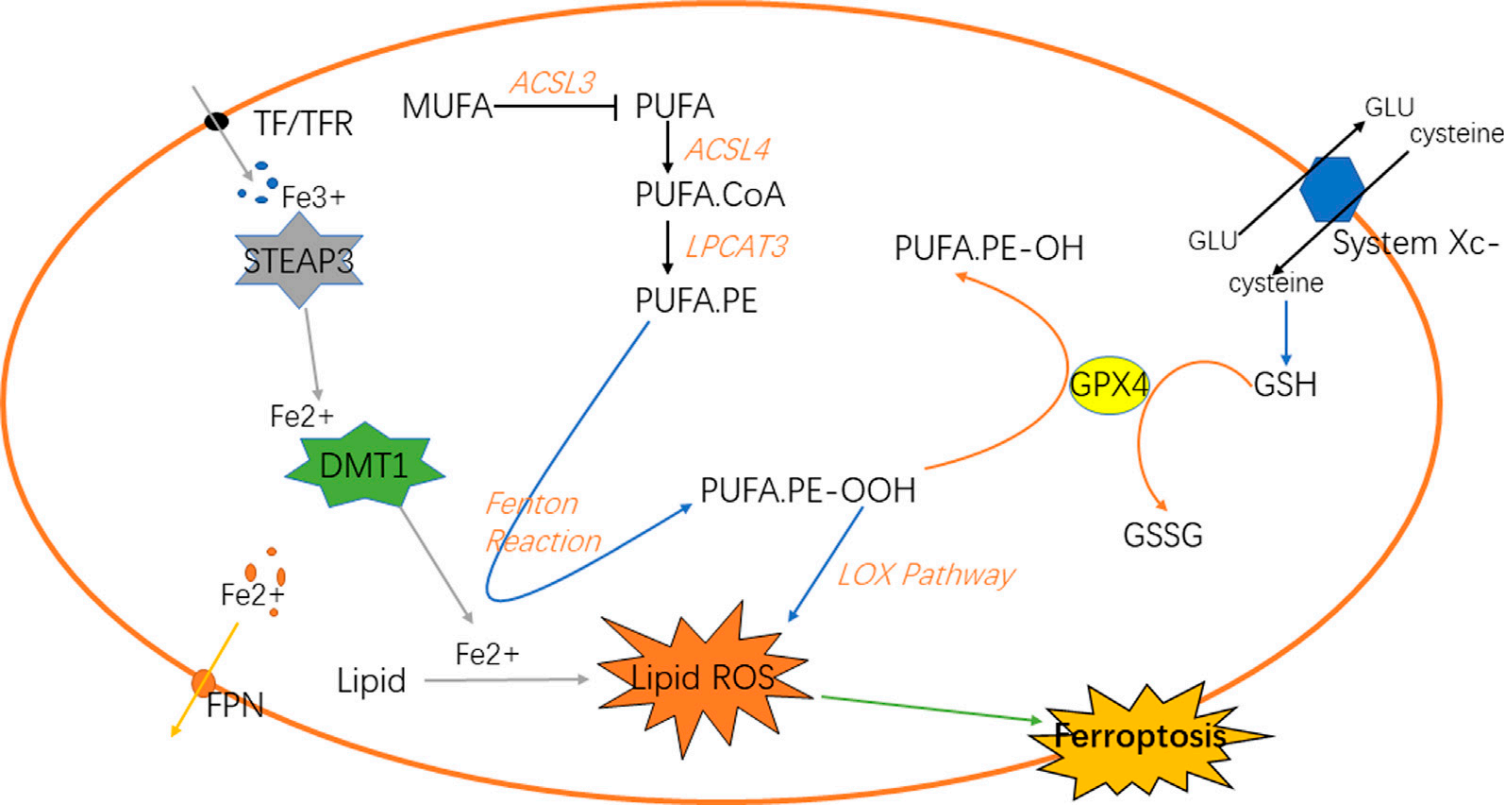
Classic signaling pathways of ferroptosis in cells.

Iron is a vital element for the survival of organisms. It is indispensable for the metabolism of many substances in the body [(Dutt et al., 2022); (Stallhofer et al., 2022)]. Iron in the digestive tract is mainly absorbed in the duodenum and upper jejunum in the form of Fe^2+^ (Karaskova et al., 2021). Then, in the epithelial cells of small intestinal mucosa, Fe^2+^ is oxidized to Fe^3+^, and part of Fe^3+^ in the blood is further bound to the transported by transferrin (TF) and receptor (TFR) on the cell membrane and transported into the cell [(Piskin et al., 2022); (Zhao et al., 2022)], which reacts with the metal reductase Six-transmembrane epithelial antigen of prostate 3 (STEAP3) in the endoplasmic reticulum to form Fe^2+^; Fe^2+^ is transported to cells through transferrin receptor protein 1 (TFR1). The body regulates the storage of iron ions through the expression of ferritin and its related genes according to its own needs, such as ferritin heavy chain 1 (FTH1), ferritin light chain (FTL) and heat shock protein B1 (HSPB1) (Yang et al., 2022a); HSPB1 inhibits the expression of TFR1, thereby reducing the intracellular iron concentration. Therefore, overexpression of HSPB1 will inhibit ferroptosis [(Yuan et al., 2022); (Liu et al., 2021)]. In addition, the expression of FTL and FTH1 is regulated by iron responsive element binding protein 2 (IREBP2), and the overexpression of IREBP2 will inhibit iron death induced by erastin as well. The biological toxicity of iron ions is mediated by Fenton reaction, which transforms Fe^2+^ into Fe^3+^ and generates hydroxyl radicals, oxidative proteins et cetera (Zhang et al., 2022a; Gong et al., 2022). Knockout of *Tf* gene or down-regulation of TF can inhibit iron overload and cell apoptosis. Autophagy can regulate the amount of transferrin and lipid in cells to regulate iron metabolism, and ultimately affect iron death sensitivity, while ferritin selective autophagy can also regulate fine ferroptosis sensitivity. Other proteins may as well affect iron sensitivity in iron metabolism (Zhu and Fan, 2021) (Figure 1).

## Lipid metabolism and reactive oxygen species accumulation regulate ferroptosis

Under normal circumstances, the substance in the organism is in a dynamic change process. When the body is disturbed by external or internal irresistible stimulus, the balance of homeostasis will be impaired (Badr et al., 2020). For example, oxidoreduction reaction disbalance and production of reactive oxygen species (ROS) in GBM (Reichert et al., 2020), the normal physiological conditions are disturbed, and the antioxidant capacity of cells will be relatively reduced (Burić et al., 2019; McNamee et al., 2021). A series of chain reactions of peroxidation will lead to the accumulation of lipid peroxidation in cells. Lipid peroxidation is a key step in ferroptosis (Lee and Roh, 2022), which can be divided into the synthesis of phospholipids with polyunsaturated fatty acids (PUFAs) as the substrate and the two peroxidation reactions. PUFAs are the key substances in lipid peroxidation (Yang et al., 2016). Firstly, PUFA.coenzyme A (PUFA.CoA) was synthesized with PUFAs under the action of acetyl CoA synthetase long-chain family member 4 (ACSL4) and then PUFA-phospholipids (PUFA-PL) was generated under the action of lysophospholipidacyltransferase 3 (LPCAT3) and incorporated into the cell components. PUFA-PL will participate in the next series of chain reactions as the substrate of peroxidation. Next, it can be divided into two ways to generate free radicals with strong oxidation capacity. Fenton reaction and lipoxygenase pathway will generate a large number of hydroxyl radicals and oxygen radicals (Xing et al., 2021). These free radicals will attack PUFA-PL, forming a cycle of peroxidation. Because cells are in a state of imbalance, this reaction will be terminated only when the substrate is exhausted. As one of the components of cells, its large consumption will inevitably lead to the damage of cell structure. In the lipoxygenase pathway (Tomita et al., 2019), 15-lipoxygenase (15-LOX) first combines with phosphatidylethanolamine binding protein 1 (PEBP1), and then oxidizes PUFA on the cell membrane to PUFA.phosphatidylethanolamines-OOH (PUFA.PE-OOH), thus causing ferroptosis (Kagan et al., 2020; Anthonymuthu et al., 2021; Mikulska-Ruminska et al., 2021). In addition to the end-products that will destroy the normal structure of cells and generate lipid peroxidation (Chng et al., 2021), the above peroxidation reactions will also produce some cytotoxic active aldehydes, which will attack phospholipids, proteins and even nucleic acids in cells (Gao et al., 2018; Liu et al., 2020; Zhao et al., 2020). NF-E2-relatedfactor2 (NRF2) plays an important role in regulating the homeostasis of cells and a key factor in oxidoreduction reaction (Zhang et al., 2016; Fan et al., 2017). Relevant studies have shown that when NRF2 is up-regulated in tumors, it is closely related to the poor prognosis of primary malignant brain tumors (Chew et al., 2021); the NRF2-Keap1 system regulates the expression of human antioxidant proteins, which is a typical antioxidant reaction pathway. Fe^2+^ is regulated by NRF2. Normally, it is inactive. When the intracellular peroxide increases, it will stimulate NRF2 to activate the downstream antioxidant enzymes to inhibit the oxidation reaction. Glutathione peroxidase 4 (GPX4) is also a key factor in the antioxidant system, which converts the lipid peroxide into non-toxic aliphatic alcohol and reduces H_2_O_2_ (Shin et al., 2018; Wu et al., 2022).

Lipid peroxidation in ferroptosis is regulated by several regulatory axes. Among them, GPX4 is recognized as the key regulatory target (Hayashima and Katoh, 2022). GSH and GPX4 in the signal axis of cystine/glutathione/glutathione peroxidase 4 can associate with each other to reduce PUFA-PL-OOH to PUFA-PL-OH, resultantly preventing the continuation of ferroptosis (Li et al., 2022a). Cystine is one of the components of glutathione, which requires the reverse transporter system Xc- to transport from outside the cell. Therefore, this transporter is also an important site for regulating ferroptosis. The signal axis of ferroptosis suppressor protein 1 (FSP1) and coenzyme Q10 (CoQ10) is also one of the regulatory sites of ferroptosis. When the GPX4 system is lacking (Doll et al., 2019), the increased expression of FSP1 can up-regulate the NADH dependent CoQ10 reduction reaction, so that the oxygen free radicals in the peroxidation cycle reaction caused by it are reduced to prevent ferroptosis (Santoro, 2020; Yang et al., 2022b). GTP cyclohydrolase 1 (GCH1)/tetrahydrobiopterin (BH4)/dihydrofolate reductase (DHFR) signal axis, in which the overexpression of GCH1 can selectively protect membrane phospholipids with two PUFA tails from peroxidation, preventing ferroptosis from occurring as well (Hu et al., 2022; Jiang et al., 2022) (Figure 1).

## Immunotherapy for glioblastoma

Iron is pivotal for cell survival. Cancer cells are more prone to undergo ferroptosis than normal cells, and oxidized membrane lipids on ferroptotic cells can mediate the phagocytosis of macrophages to keep immune response. In addition, immune detection point inhibitor treatment may make cancer cells sensitive to ferroptosis (Shi et al., 2022b), it is expected to overcome chemical resistance and strengthen the death of immunogenicity cells. Enhanced ferroptosis was shown to induce activation and infiltration of immune cells, but to weaken the cytotoxicity of anti-tumor cells. It was found that tumor-associated macrophages were involved in ferroptosis-mediated immunosuppression (Liu et al., 2022a), which provides a new vision for immunotherapy for GBM (Yu et al., 2022).

Inhibition of asparagine-linked glycosylation 3 (ALG3) stimulates cancer cell immunogenic ferroptosis to potentiate immunotherapy. ALG3 is an a-1, 3-mannosyltransferase involved in protein glycosylation in the endoplasmic reticulum. Liu et al. reported that inhibition of ALG3 would induce defects in post-translational N-linked glycosylation modification and lead to excessive lipid accumulation through sterol regulatory element binding proteins (SREBPs)-dependent adipogenesis in cancer cells. Lipid peroxidation mediated by N-linked glycosylation deficiency induces the immunogenic ferroptosis of cancer cells and promotes the pro-inflammatory microenvironment, thus enhancing the anti-tumor immune response (Liu et al., 2022b).

Ir(III) complex containing a ferrocene-modified diphosphine ligand that localizes in lysosomes. Under the acidic environments of lysosomes, Ir(III) can effectively catalyze a Fenton-like reaction, produce hydroxyl radicals, induce lipid peroxidation, down-regulate GPX4, resulting in ferroptosis, and thus inhibit tumor cell growth (Wang et al., 2022).

**FIGURE 2:**
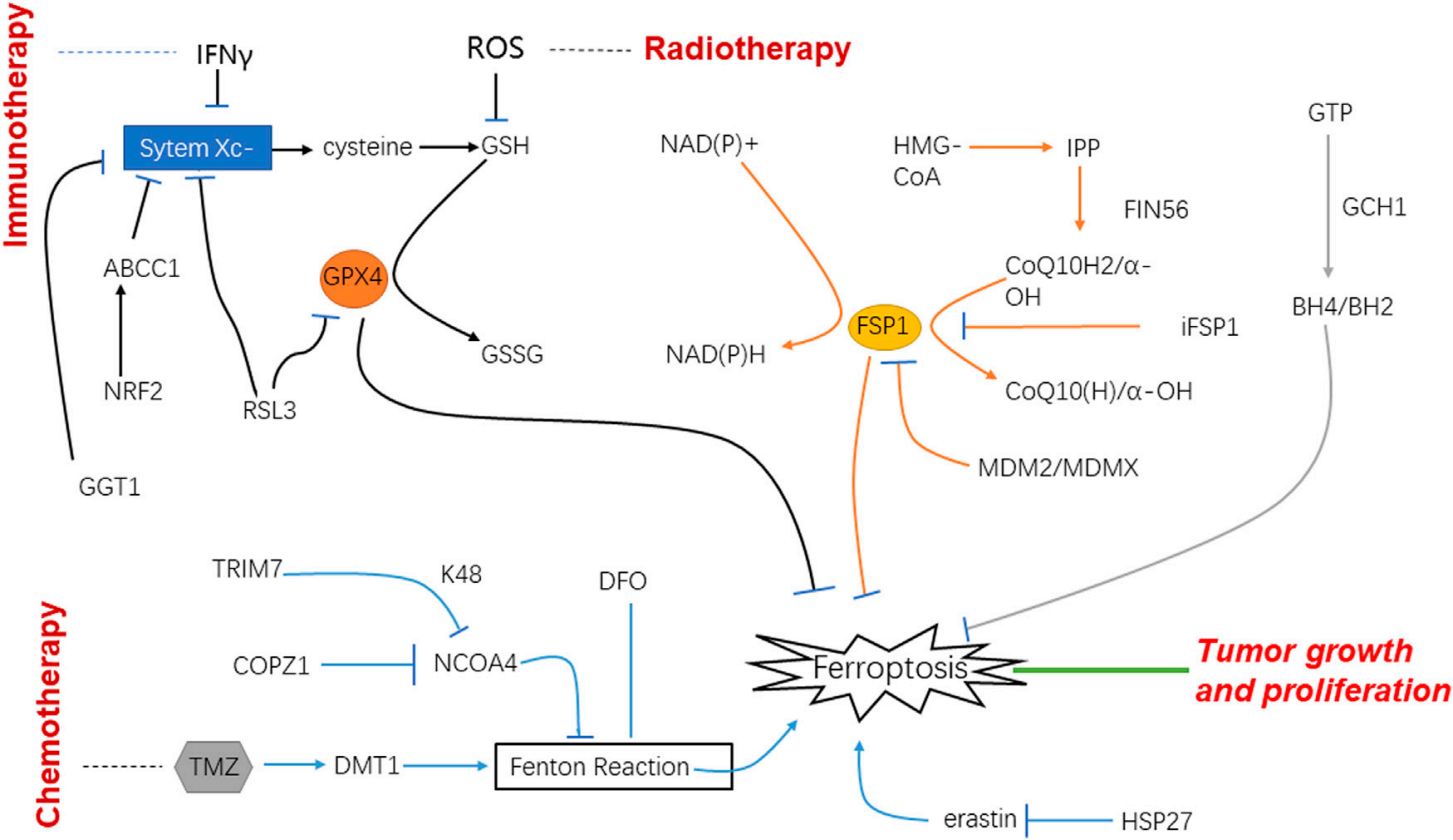
Schematic of mechanisms of ferroptosis and glioblastoma under the treatment of immunotherapy, radiotherapy and chemotherapy.

In recent years, immunotherapy has raised fervent attention. One strategy is to activate lymphocytes that can recognize the tumor cells specifically and induce the cancer cells’ death by releasing perforin and granzyme. Accordingly, relevant studies pointed out that active CD8^+^ T cells release IFNγ that down-regulate the expression of Solute carrier (SLC) family 7 member 11 (SLC7A11) and SLC family 3 member 2 (SLC3A2), thereby inhibiting the absorption of cystine and enhancing lipid peroxidation and ferroptosis of tumor cells (Figure 2).

## Radiotherapy for glioblastoma

Ionizing radiation can produce a lot of free radicals in irradiated cells. These free radicals are the factors that cause side effects when ionizing radiation is used to treat diseases. Among them, lipid peroxidation is an important influence of ionizing radiation on cell membranes, which is one of the key targets of radiation. On the other hand, lipid peroxidation is an important metabolic pathway for ferroptosis. Therefore, the effect of ionizing radiation on tumor cells should be related to the ferroptotic process to a large extent (Agrawal and Kale, 2001). It was found that after radiotherapy, the tumor cells showed typical morphological changes of ferroptosis such as changed density of mitochondria, shrunk cell membranes, and decreased mitochondrial cristae. It indicates that radiotherapy can increase the expression of key enzymes by generating a large number of ROS, thus promoting lipid peroxidation, and ultimately leading to ferroptosis (Zhang et al., 2022b). CuS@mSiO_2_@MnO_2_ nanocomposite, as a radiosensitizer that can effectively exhaust GSH was used *in vitro*; it uses up GSH and induces ferroptosis and apoptosis. The experiment performed *in vivo* also showed that tumor cells were damaged. Therefore, it is speculated that radiotherapy may induce ferroptosis after the depletion of GSH to play its role in the treatment of tumors (Li et al., 2021) (Figure 2).

## Chemotherapy for glioblastoma

Temozolomide (TMZ) is the first-line drug for clinical chemotherapy for GBM in recent decades. Although it is the most effective drug for GBM for now, resistance to it especially in recurrent GBM can be noted very common. Drug resistance is one of the most significant factors which bring about a poor prognosis. NRF2 is an important transcription factor involved in chemotherapy resistance, and it can play a key role in inducing ferroptosis through GSH regulation. According to the experimental data, high-level of NRF2 leads to the resistance to TMZ therapy for GBM through the up-regulation of ATP-binding cassette subfamily C member 1 (ABCC1) which is the target of NRF2 and able to antagonize ferroptosis. For this rationale, ferroptosis induction may be an important treatment strategy to reverse the drug resistance of GBM with high NRF2 and ABCC1 expression (de Souza et al., 2022). Most chemotherapy medicines exert efficacy on tumor cells through signaling transduction which are bound up with oxygen-free radicals and ROS; under the circumstance, ferroptosis will play important role in the therapy of malignant tumors. Nowadays, TMZ is a new medicine for GBM and considered the most effective one, and thus widely used for patients in clinical practice (Zhou and Ma, 2022). There have been *in vitro* studies using TG905 cells showing that the divalent metal transporter 1 (DMT1) and ROS in GBM increases when treated with TMZ, whereas GPX4 is decreased (Su et al., 2022), in the meantime it observes an iron-independent cells death. Thus, we have good reason to believe that TMZ can induce the ferroptosis by targeting the expression of DMT1 in GBM cells and inhibiting tumor cells growth (Song et al., 2021).

It is reported that heat shock protein 27 (HSP27) is the new regulator of ferroptosis in tumor cells (Liu et al., 2021); GBM cells are protected by overexpression of HSP27 to escape from ferroptosis that is induced by erastin (Yuan et al., 2022). Therefore, HSP27 can be a regulatory spot of ferroptosis and used as a potential therapy target for GBM.

Clinical studies have shown that programmed cell death one (PD-1)/programmed cell death ligand one (PD-L1) have low efficacy on GBM due to low immunogenicity. In the experiment, Fe_3_O_4_-siPD-L1@M-BV2 increases the siPD-L1 and Fe^2+^ of the drug-resistant GBM in mice tissue. Fe_3_O_4_-siPD-L1@M-BV2 is associated with ferroptosis and immune activation, inhibiting the growth of drug-resistant GBM (Liu et al., 2022c).

Chen et al. reported that IONP@PTX can inhibit cell migration and invasion after incubating with cells. They made the conclusion that the level of iron ions, ROS and lipid peroxidation in cells is increased, suggesting that IONP@PTX might affect GBM through ferroptosis. More importantly, the influence of IONP@PTX on GBM can be regulated by other external factors such as 3-methyladenine (3-MA) and rapamycin. Another advantage is that IONP@PTX has no obvious toxic effect on GBM xenotransplantation mice, which may make IONP@PTX a potential treatment for GBM with high safety based on ferroptosis (Chen and Wen, 2022).

Some scholars reported that NF-κB activation protein (NKAP) knockout will increase the level of lipid peroxidation in naive T cells and induce cell death in colon cancer cells. Another experiment showed the consistent results that knockout of NKAP induces the death of GBM; silencing NKAP increases the sensitivity of cells to iron death inducers, and exogenous overexpression of NAKP can positively regulate an iron death defense protein, SLC7A11 to reduce the sensitivity of cells to iron death inducers. RNA and protein immunoprecipitation can prove that NKAP and N6-methyladenosine (m6A) interact on SLC7A11 transcription. Thus, inhibiting NKAP expression or knocking out its gene will increase the level of ferroptosis in cells, and may become a potential therapeutic direction to the treatment of GBM (Sun et al., 2022).

Ferroptosis is mainly caused by an imbalance of ROS and lipid peroxidation. One of the processes of lipid peroxidation can be inhibited by GPX4. In consequence, blocking the expression of GPX4 or reducing its production will greatly increase the degree of ferroptosis. γ-glutamine transferase 1 (GGT1) can inhibit the formation of the substrate in the process of synthesizing GPX4. Given this, GGT inhibitor is a potential treatment scheme for GBM, and it will be more effective if it is supplemented with iron death inducers (Hayashima and Katoh, 2022).

Based on the analysis of existing data, the overexpression of coatomer protein complex subunit zeta 1 (COPZ1) is related to the increase in tumor grade and poor prognosis of GBM patients. Via immunohistochemistry and western blot analysis, it is noted that the expression of COPZ1 protein in GBM was significantly higher than that in normal tissues. Knockout of *Copz1* gene using iRNA could inhibit the growth of GBM *in vitro*, but also lead to a series of intracellular metabolic disorders, including the imbalance of iron metabolism. Therefore, COPZ1 can be a key regulatory point of ferroptosis, and become a potential therapeutic target for GBM (Zhang et al., 2021b).

Dihydroartemisinin (DHA) has the advantages of selective cytotoxicity and low drug resistance. These characteristics make DHA one of the new research directions of anti-tumor therapy. Through the effect of DHA on normal cells and GBM, it is found that GPX4 in tumor cells is significantly reduced, ROS and peroxidative lipids are increased, and these effects can be reversed by using ferroptosis inhibitors, which demonstrates DHA can inhibit tumor growth by changing the intracellular GPX4 to cause ferroptosis. DHA is one of the potential drugs for GBM (Yi et al., 2020) (Figure 2).

## Ferroptosis in glioblastoma

The main component of brain tissue is lipid. At present, there is no effective treatment for GBM, and the recurrence rate and mortality rate are nearly 100% (Mitre et al., 2022). Currently, ferroptosis as the research field remains many unrevealed areas, which may become a potentially effective treatment scheme. The brain is more vulnerable to oxidative stress than other tissues, because the activity of antioxidant enzymes is low and the content of PUFAs is high, which makes it prone to lipid peroxidation (Rao et al., 2000). The disorder of lipid metabolism is the key link of ferroptosis. It was found that expression of tripartite motif protein 7 (TRIM7) is more in GBM cells than in normal cells. When the TRIM7 is silent, the growth and development of the body is inhibited, but the level of ferroptosis is increased; while the TRIM7 is overexpressed, it can promote the growth and development of the body and inhibit the death of GBM cells. It can be concluded that when the ferroptosis level is inhibited, the death of GBM cells is also reduced, and there is a positive correlation between them. This experiment also indicates that when TRIM7 is missing, GBM is sensitive to the treatment of TMZ. As a potential treatment scheme for GBM, it is uncertain whether the sensitivity of TRIM7 to ferroptosis will make the treatment of TMZ more effective (Li et al., 2022b). Some experiments also found that the use of iron inducers can increase the sensitivity of TMZ. TMZ is a first-line drug for GBM (Song et al., 2021), and it is clear that its mechanism is to cause base pair mismatch, leading to cell apoptosis. However, several reports suggest that, it can activate nuclear factor NRF2 and transcription factor 4 (ATF4) at the same time to inhibit iron death. To sum up, ferroptosis participated not only in the sensitivity of TMZ in the treatment of GBM, but also in the formation of drug resistance (Chen et al., 2017; Hu et al., 2020; Li et al., 2022b; de Souza et al., 2022). Iron overload is another key link of ferroptosis. The high degree of malignancy of tumors is characterized by rapid growth and proliferation and strong invasiveness. Genes and proteins related to growth and development are overexpressed, such as poly(C)-binding protein 2 (PCBP2), DMT1, STEAP3, FTH and FTL, which can change iron storage capacity (Cheng et al., 2018); the expression of TF and TFR also increased significantly. A large amount of free iron would be transported and absorbed into the cells through selective endocytosis, and excessive iron would increase lipid peroxidation accordingly. In GBM cells with overloaded iron and rich PUFAs, plenty of peroxides can rapidly accumulate and lead to ferroptosis (Park et al., 2019). STEAP3 can promote TFR1 expression and increase cell iron content by activating STAT3-forkhead box protein M1 (FOXM1) axis (Sendamarai et al., 2008; Isobe et al., 2011), thus inducing epithelial mesenchymal transformation (EMT) in GBM, which is a route for GBM invasion and metastasis (Chang et al., 2017; Terry et al., 2017).

## Expectation

Ferroptosis is a newly discovered iron dependent programmed cell death in recent years, which is different from apoptosis, necrosis and necroptosis. It has a unique mechanism of occurrence and effectiveness. It will be a potential therapeutic scheme in tumor treatment, and more and more targeted ferroptosis therapies are under study. Relevant pharmaceutical industries are also actively exploring the specific mechanism of iron death, trying to link it with cancer treatment to find a breakthrough. So far, although ferroptosis has made some theoretical achievements and curative effect in animal experiments, it has not made virtual progress. As aforementioned, some scholars proposed even contradictory conclusions. Some pointed out that TMZ treatment could strengthen ferroptosis and might be one of the mechanisms of tumor cell killing, whereas other researchers opposed this kind of conclusion and observed the opposite that TMZ might inhibit ferroptosis to result in drug resistance. This is an interesting phenomenon, and we speculate that TMZ treatment indeed up-regulates ferroptosis and causes the death of tumor cells. However, at the late stage of the course, the GBM cells may induce numerous ferroptotic inhibitors and the ferroptosis is significantly suppressed. This is worthy of being studied in the future. GBM, as one of the most common primary malignant tumors in adults, has not only a high recurrence rate but also an approximately mortality rate of 100%. Increasing evidence indicates that ferroptosis plays a certain role in immunotherapy, radiotherapy and chemotherapy for GBM. However, the research on its regulation and the mechanism of ferroptosis treatment has not made significant progress. It is still necessary to take persistent efforts to clarify the mechanism of the relationship between ferroptosis and GBM, to provide new ideas for the treatment, and in the meantime to bring up prevention or early diagnosis methods.
